# Does controlled ovarian hyperstimulation in women with a history of borderline tumor influence recurrence rate?

**DOI:** 10.1007/s00404-023-07103-8

**Published:** 2023-09-26

**Authors:** Han Gao, Wei Wei, Yibing Li, Heng Wei, Ning Wang

**Affiliations:** 1 Department of Obstetrics and Gynecology, Affiliated Hospital of Inner Mongolia Minzu University, Tongliao, China; 2 Department of Obstetrics and Gynecology, The Second Affiliated Hospital of Dalian Medical University, No. 467 Zhongshan Road, Shahekou District, Dalian City, 116027 Liaoning Province China; 3 Department of Obstetrics and Gynecology, Shengjing Hospital of China Medical University, Shenyang, China

**Keywords:** Borderline ovarian tumors, Assisted reproductive technology, Fertility preservation, Recurrence rate, Overall survival

## Abstract

**Purpose:**

To determine the recurrence rate in the women with controlled ovarian hyperstimulation after a history of borderline ovarian tumors (BOT).

**Methods:**

This was a retrospective analysis of 275 patients with BOT undergoing surgery for fertility preservation in our hospital between 2001 and 2017. Cases were divided into an assisted reproductive technology (ART) treatment group (n = 15) and a non-ART treatment group (n = 260). We compared the recurrence rate, survival rate and pregnancy outcomes between these two groups.

**Results:**

The ART group had a higher recurrence rate (33.33% vs. 10.80%, P = 0.023). Survival analysis indicated that the recurrence time in patients undergoing ART was significantly shorter (P = 0.026). A low pregnancy rate before diagnosis, and high intraoperative blood loss, were associated with postoperative ART treatment (P < 0.05). Multivariate analysis showed that ART treatment and bilateral lesions both significantly increased the risk of recurrence (P < 0.05). The pathological type of recurrent tumors was often the same as the initial tumor.

**Conclusion:**

The postoperative use of ART in patients with BOT significantly increased the recurrence rate, but does not significantly affect the overall survival rate of patients. Therefore, ART in such patients should be individualized, and close follow-up is necessary after ART.

## What does this study add to the clinical work

**Table:** 

The postoperative use of ART in patients with BOT significantly increased the recurrence rate. So we recommend that ART in such patients should be individualized, and closely follow-up is necessary after ART.	

## Introduction

Borderline ovarian tumors (BOT) are a type of low malignant potential (LMP) tumor that can seriously damage the lives and health of women. First described in 1929, BOT is defined as a type of tumor that differs from ovarian epithelial cancer (EOC) [1]. Indeed, BOT was once identified as a precursor of EOC, and the principle of treatment was the same as for ovarian epithelial cancer.

As research into BOTs has intensified, authors identified that biological behavior was critical in an independent individual and defined whether ovarian epithelial tumors remained benign or became malignant; these tumors were first staged by the International Federation of Gynecology and Obstetrics (FIGO) in 1970 [2].

The clinical features of BOT are also different from EOC. For example, these tumors were more common than ovarian cancer in women of childbearing age. A key consideration here is that young women can often require fertility preservation, with an average onset between 30 and 50 years of age. BOT constitutes between 10 and 15% of all ovarian epithelial tumors, most of which are serous or mucinous tumors [3]. Furthermore, patients with BOT present at earlier clinical stage in the initial diagnosis, while 70–80% are diagnosed at early FIGO stage (II) [4]. Some previous studies reported that BOT patients are associated with a better prognosis and a 5-year survival rate exceeding 95% [5]. Finally, cases of BOT are generally not associated with interstitial infiltration [6].

Previous studies have reported that patients with ovarian epithelial borderline tumors after conservative surgery can spontaneously become pregnant [7–9], although many of these patients suffer from infertility and require infertility treatment. However, the influence of infertility treatment on the development of BOT has not yet been fully elucidated. While some existing studies reported an association between the occurrence of BOT and the use of ovulation inducing drugs [10–14], others did not [15, 16]. Indeed, previous research indicated that patients with BOT undergoing ART were four times more likely to experience tumor growth [17]. At present, only a few studies have investigated BOT patients after conservation treatment who then undergo ART procedures. The purpose of our study was to investigate the effect of ART in BOT patients who previously underwent surgery for fertility preservation.

## Materials and methods

The study group was composed of 275 patients with BOT undergoing fertility preservation surgery in the SHENG JING Hospital between 2001 and 2017; 49 patients were lost to follow-up. The 275 patients were divided into an ART treatment group (n = 15) and a non-ART treatment group (n = 260). Of these 275 patients, 33 suffered recurrence and 242 had no recurrence. Our inclusion criteria were as follows: age less than 46 years, normal menstrual cycles and no history of infertility before surgery, no other prior pelvic surgery, patients undergoing fertility preservation surgery. All patients were diagnosed with BOT according to postoperative paraffin pathology with two or more pathologists. All procedures performed in studies involving human participants were in accordance with the ethical standards of the institutional and/or national research committee and with the 1964 Helsinki declaration and its later amendments or comparable ethical standards. We obtained informed consent from all participants or, if subjects are under 18, from their legal guardians.

The aim of conservative surgery is to preserve the uterus and one or part of the ovary. An additional surgical stage is often required, including peritoneal washing, bilateral pelvic lymphadenectomy, omentectomy, appendectomy and multiple peritoneal biopsy. After conservative surgery, some patients suffer from infertility and require treatment. There are several causes of infertility, including low pregnancy rates prior to surgery and infertility caused by conservative surgical treatment, including pelvic adhesions and residual ovarian tissue reduction. The use of certain methods to regulate ovulatory function in the ovaries is a key aspect of ART. And there are many forms of controlled ovarian stimulation (COS), including agonist protocols [gonadotropin releasing hormone agonist (GnRH-a)/gonadotropin(Gn)/human chorionic gonadotropin (hCG) protocol], antagonist protocols [Gn/GnRH-anti/hCG protocols] and microstimulation protocols. In addition, the agonist protocol can feature either a long protocol and a short protocol. For further information relating to the drugs used for ovulation induction, please refer to the work of Ron-El et al. [18].

Following conservative surgery, patients undergoing follow-up require gynecological examinations, examinations of tumor markers, and vaginal ultrasonography every 3 months for 2 years, and every six months thereafter. We obtained survival, recurrence and pregnancy related data for all patients throughout the follow-up period.

Analysis was performed using SPSS version 19.0 statistical software. The χ^2^ test was used to compare numerical data between groups, while the t-test was used to compare measurement data. Survival rate was calculated using the Kaplan–Meier method and statistical differences were compared using the log-rank method, the Breslow method and The arone-Ware method. The Cox proportional hazard model was used for multivariate analysis and P < 0.05 was considered statistically significant. For further methodsplease refer to the work of Jiaqi liu et al. [19].

## Results

### Normal information

During our study period (2001–2017), we identified 324 patients with a pathological diagnosis of an ovarian epithelial borderline tumor who were undergoing fertility preservation surgery. A total of 49 patients were lost to follow-up. Thus, 275 patients were included in the final analysis. The median follow-up time was 49 months. We divided the 275 patients into an ART treatment group (n = 15) and a non-ART treatment group (n = 260). In total, 33 patients suffered recurrence and the overall recurrence rate was 12.00%. The recurrence rate of the ART group was 33.33% (n = 5), while that of the non-ART treatment group was 10.80% (n = 28; P = 0.023).

The clinical variables are shown in Table 1. There were no significant differences between the two groups in terms of age, surgical approach, bilateral and unilateral lesions, surgical complications or pathological types (P > 0.05). We found that more patients undergoing surgical staging accepted ART treatment (40%), compared to those who did not undergo surgical staging (18.8%); however, there was no significant difference between the two groups (P > 0.05).

**Table 1:** Clinical and pathological features of patients with fertility preservation surgery (enumeration data)

	Non-IVF group	IVF group	P
	n (%) (n = 260)	n (%) (n = 15)	
Age at diagnosis (years)			
≤ 35	182 (70.0)	11 (73.3)	0.521
> 35	78 (30.0)	4 (26.7)	
Pathological type			
Serous	137 (52.7)	10 (66.7)	0.522
Mucinous	118 (45.4)	4 (26.7)	
Other	5 (1.9)	1 (6.7)	
Lateral			
Unilateral	229 (88.1)	14 (93.3)	0.459
Bilateral	31 (11.9)	1 (6.7)	
FIGO stage			
I	254 (97.7)	15 (100.0)	0.576
II	4 (1.5)	0 (0.0)	
III	2 (0.8)	0 (0.0)	
IV	0 (0.0)	0 (0.0)	
Surgical approach			
Laparotomy	164 (63.1)	9 (60.0)	0.505
Laparoscopy	96 (36.9)	6 (40.0)	
Surgical type of ovaries			
Unilateral cystectomy	64 (24.6)	5 (33.3)	0.402
Unilateral Salpingo-oophorectomy	78 (30.0)	0 (0.0)	
Bilateral cystectomy	33 (12.7)	3 (20.0)	
Unilateral Salpingo-oophorectomy + cystectomy	85 (32.7)	7 (46.7)	
Surgical staging			
Yes	49 (18.8)	6 (40.0)	0.056
No	211 (81.2)	9 (60.0)	
Recurrences			
No	232 (89.2)	10 (66.7)	**0.023**
Yes	28 (10.8)	5 (33.3)	

Rates of recurrence and associated clinical variables are shown in Table 2. Unilateral ovarian cysts were identified in 147 patients and 128 patients had bilateral ovarian tumors. Different forms of surgery were carried out, including unilateral salpingo-oophorectomy (USO, n = 78 patients), unilateral cystectomy (UC, n = 69 patients), unilateral salpingo-oophorectomy + cystectomy (USO + CC, n = 92 patients), and bilateral cystectomy (BC, n = 36 patients). Eight of the 69 patients (11.59%) who had UC treatment, and nine of the 78 patients (11.54%) who had USO treatment experienced tumor recurrence. The recurrence rate of patients with BC (16.67%) was higher than those with USO + CC (10.87%), although this difference was not statistically significant (P > 0.05). In total, 33 patients with disease recurrence showed the same form of pathological type as when they were treated by conservative management for their initial tumor. Disease recurred in the unilateral ovary. Other factors had no significant effect on recurrence, including surgical staging, pathological type and surgical approach.

**Table 2:** Risk factors of recurrence in patients with fertility preservation surgery (enumeration data)

	Non-Recurrence	Recurrence	P
	n (%) (n = 242)	n (%) (n = 33)	
Age at diagnosis (years)			
≤ 35	167 (70.0)	26 (78.8)	0.313
> 35	75 (30.0)	7 (21.2)	
Pathological type			
Serous	127 (52.5)	20 (60.6)	0.351
Mucinous	109 (45.0)	13 (39.4)	
Other	6 (2.5)	0 (0.0)	
Lateral			
Unilateral	216 (89.3)	27 (81.8)	0.337
Bilateral	26 (10.7)	6 (18.2)	
FIGO stage			
I	236 (97.5)	33 (100.0)	0.460
II	4 (1.7)	0 (0.0)	
III	2 (0.8)	0 (0.0)	
IV	0 (0.0)	0 (0.0)	
Surgical approach			
Laparotomy	153 (63.2)	20 (60.6)	0.920
Laparoscopy	89 (36.8)	13 (39.4)	
Surgical type of Ovaries			
Unilateral cystectomy	61 (25.2)	8 (24.2)	0.846
Unilateral Salpingo-oophorectomy	69 (38.5)	9 (27.3)	
Bilateral cystectomy	30 (12.4)	6 (18.2)	
Unilateral Salpingo-oophorectomy + cystectomy	82 (33.9)	10 (30.3)	
Surgical staging			
Yes	51 (21.1)	4 (12.1)	0.330
No	191 (78.9)	29 (87.9)	

Table 3 shows that there was no significant difference between the two groups in terms of the age at diagnosis, operative time, and hospital stay. However, the pregnancy rate before BOT in the non-ART treatment group was significantly higher than that in the ART treatment group (0.64 ± 1.061 vs. 0.20 ± 0.414, P = 0.002). In other words, the pregnancy rate before BOT was low, and the chance of facing ART treatment would likely increase in the future. This was consistent with clinical observations in that people undergoing ART treatment usually have poor pregnancies and low pregnancy rates. The intraoperative blood loss of patients who underwent ART treatment was significantly higher than those who did not have ART treatment (128.00 ± 81.70 vs. 76.35 ± 80.90 mL, P = 0.03). We found that intraoperative blood loss could be attributed to ART treatment after the surgery.

**Table 3:** Clinical features of patients with fertility preservation surgery(measurement data and Risk factors of recurrence in patients with fertility preservation surgery (measurement data)

	Non-IVF group	IVF group	P	Non-recurrence	Recurrence	P
	Mean (SD)	Mean (SD)		Mean (SD)	Mean (SD)	
Age at Diagnosis (years)	32.29 (5.90)	33.33 (4.271)	0.381	32.33 (5.781)	33.42 (6.185)	0.938
Pregancy before BOT	0.64 (1.061)	0.20 (0.414)	**0.002**	0.64 (1.074)	0.48 (0.755)	0.312
Operative time (min)	116.51 (60.22)	155.67 (88.80)	0.113	116.78 (61.25)	132.33 (70.75)	0.236
Blood loss (ml)	76.35 (80.90)	128.00 (81.70)	**0.030**	76.38 (80.29)	99.55 (89.62)	0.167
Hospital stay (days)	10.58 (3.60)	11.07 (3.69)	0.629	10.59 (3.603)	10.70 (3.670)	0.886

In addition, Table 3 also shows that there was no significant difference in the age of patients at diagnosis, operative time, and hospital stay when compared between the recurrence group and the non-recurrence group. The pregnancy rate before BOT, and intraoperative blood loss, did not have a significant effect on recurrence.

### Subsistence analysis

**Fig. 1:**
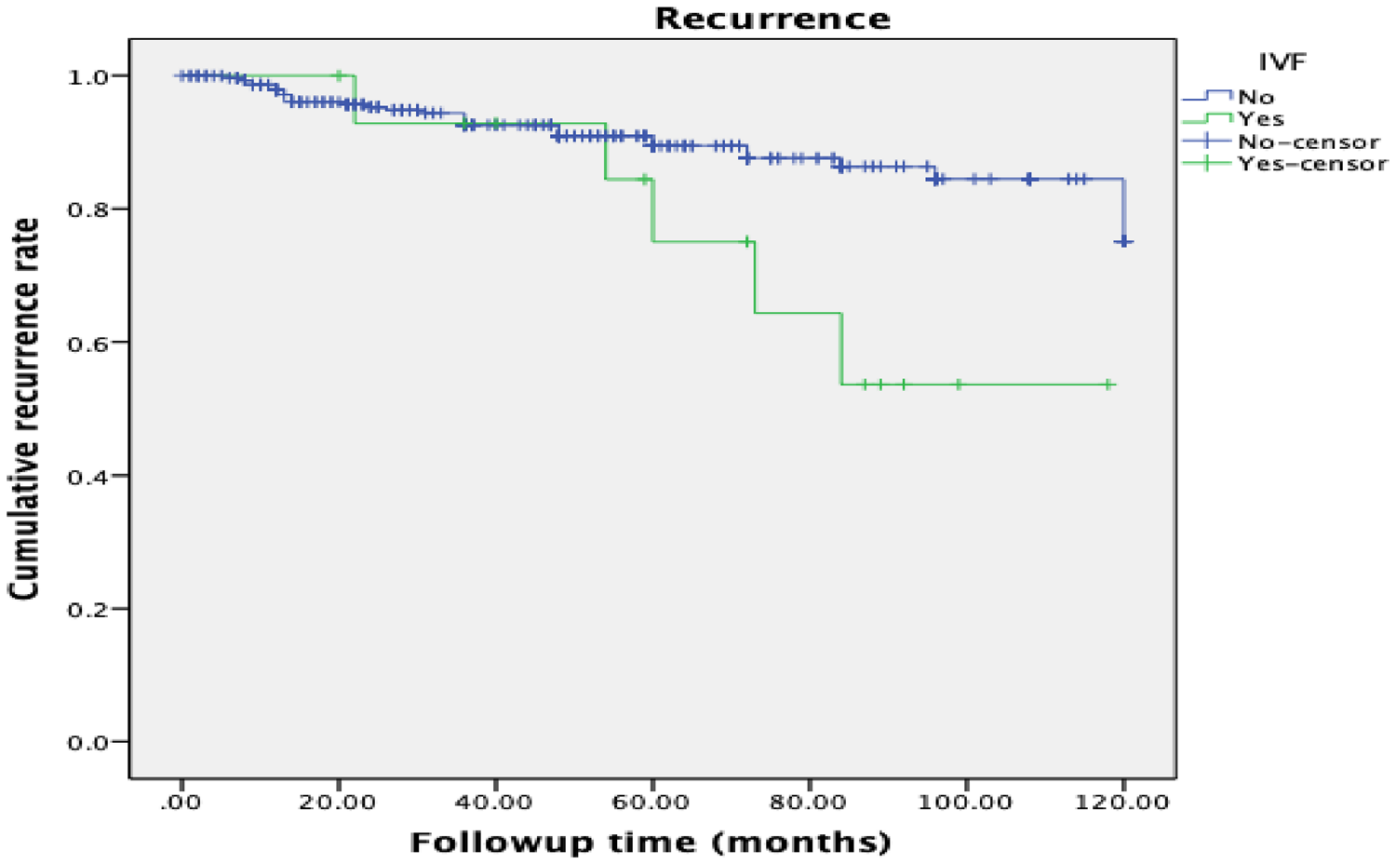
Comparison of survival in women of BOT after fertility preservation surgery. Kaplan–Meier survival curves showing the effect of IVF treatment or non-IVF treatment on disease-free survival

**Fig. 2:**
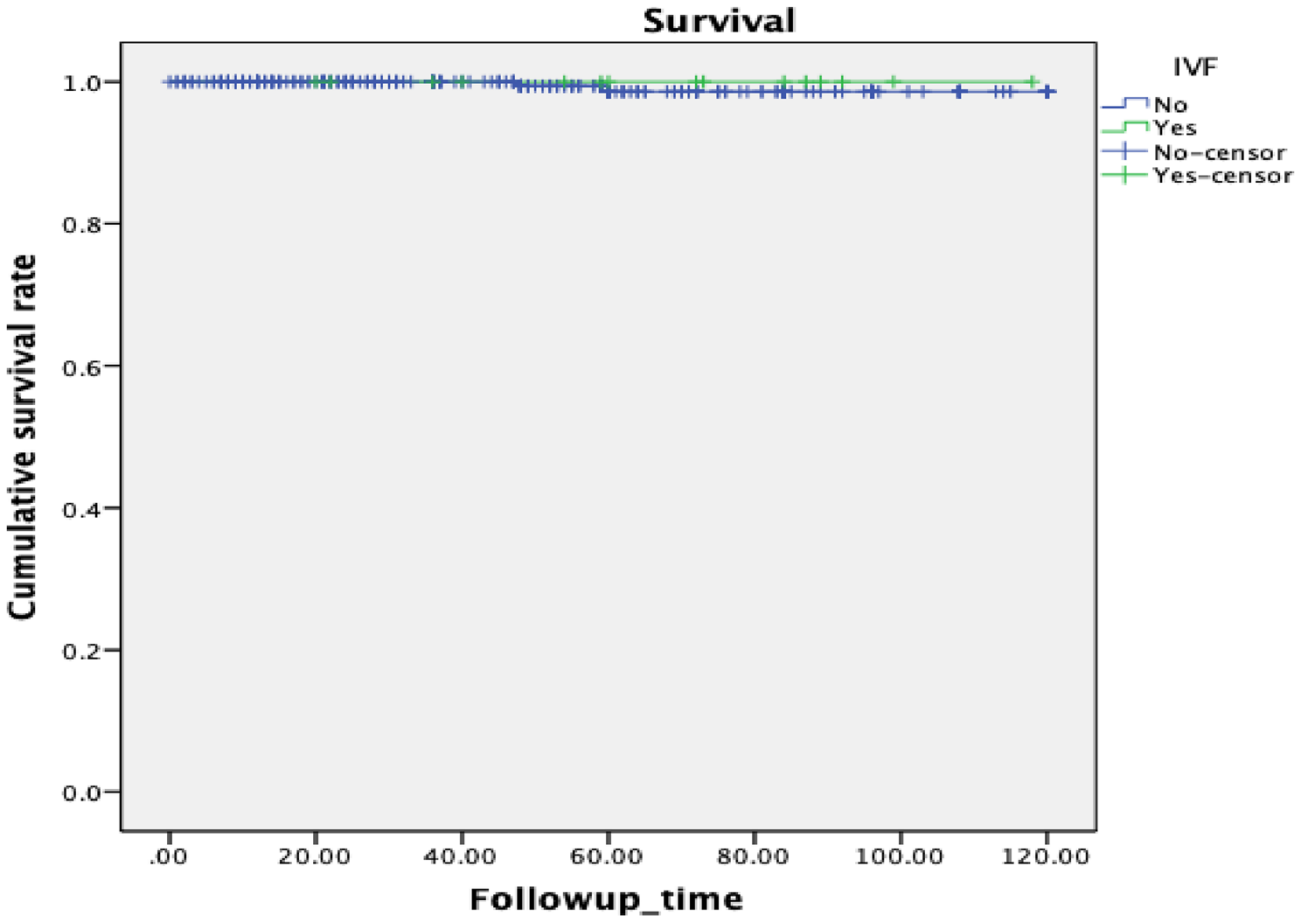
Kaplan–Meier survival curves showing the effect of IVF treatment or non-IVF treatment on total survival

We found that patients who underwent ART treatment and non-ART treatment had an average disease-free survival of 91.83 ± 9.21 and 108.76 ± 2.03 months, respectively. This difference was statistically significant (P = 0.026) (Fig. 1). Furthermore, the recurrence time of patients with ART after fertility preservation was significantly shorter, which was related to tumor recurrence. Only two patients died during our study. The statistical results suggested that there was no significant relationship between ART and patient mortality (P = 0.712) (Fig. 2). The use of ART did not affect the overall survival of patients.

### Multi-factor analysis

Table 4 shows the results of multivariate analyses of disease-free survival (DFS), ART, surgical staging, pathology type, surgical approach, FIGO stage, age and lateral lesions. We found that ART and bilateral or unilateral lesions had a significant impact on recurrence. Patients undergoing non-ART treatment tended to have a better DFS than those undergoing ART treatment (95% confidence interval [CI]: 1.273–9.869, P = 0.015). Patients with unilateral lesions tended to have a better DFS than those with bilateral lesions (95% CI: 1.563–9.366, P = 0.003).

**Table 4:** The relationship between different factors and recurrence rate in COX model analysis

	P	HR	95.0% CI
IVF	**0.015**	3.544	1.273–9.869
Surgical staging	0.064	2.839	0.940–8.572
Pathology type	0.347	0.714	0.354–1.440
Surgical approach	0.658	0.846	0.403–1.775
FIGO stage	0.980	0.000	–
Age	0.145	0.387	0.108–1.389
Lateral	**0.003**	3.827	1.563–9.366

### Assisted reproductive technology

In total, 15 patients underwent ART treatment after conservative treatment for BOT at SHENGJING hospital. Various forms of COS were carried out, including the long protocol (n = 9), short protocol (n = 2), antagonist protocol (n = 3) and micro-stimulation (n = 1). Patients receiving the long protocol had better pregnancy outcomes and a higher number of retrieved oocytes as compared to other stimulation protocols. Of the 15 patients undergoing infertility treatment, there were six patients with seven pregnancies. One patient had a twin pregnancy and delivered two healthy infants. The outcomes of the pregnancies were three miscarriages and four normal pregnancies. In the non-ART treatment group, when we removed 38 unmarried patients and 25 women who had no intention of becoming pregnant, there were 40 normal pregnancies as a result of spontaneous conception in BOT patients undergoing fertility preservation surgery. The normal pregnancy rate of the non-ART treatment group was 18.87%, while that of the ART treatment group was 26.67%. There was no significant difference in terms of pregnancy outcome between the two groups (P > 0.05). None of the patients treated with ART had been pregnant previously. We divided the 15 patients undergoing ART treatment into a recurrence group (n = 5) and a non-recurrence group (n = 10). In the recurrence group, the pathological type was identical to the primary disease. The median interval between first ART treatment and ovarian recurrence in the ART treatment group was 24 months. The median interval between initial diagnosis and recurrence in the non-ART treatment group was 25 months. There was no significant difference in terms of either follow-up time or tumor recurrence (P > 0.05). The mean duration of IVF cycles was 1.8, and the mean number of retrieved oocytes was 12. In the recurrence group, the mean number of ART cycles was two, and the mean number of retrieved oocytes was 14.00 ± 9.744. In the non-recurrence group, the mean number of ART cycles was one, and the mean number of retrieved oocytes was 10.80 ± 9.041. Four of the five patients in the recurrence group were treated with the long protocol, while the remaining patients were treated with the short protocol.

## Discussion

Ovarian borderline tumors are a type of ovarian tumor with a much better prognosis than ovarian cancer at the same clinical stage. Ovarian borderline tumors also showed cytological characteristics that can differentiate between benign and malignant tumors [3], without interstitial infiltration. Surgery is the main form of treatment. In the past decade, ovarian tumors have occurred more frequently in women under the age of 40. Previous studies have reported that fertility-sparing surgery (FSS) can increase the recurrence rate in BOT patients, but does not affect their survival rate, compared with radical surgery [20]. Most recurrences were detected during follow-up procedures, and were of an early stage. The pathological type of recurrence was the same as the initial tumor. These earlier findings were consistent with our present findings. With the creation of the new Chinese second child policy and improvements in the quality of life, the younger age of patients and the good prognosis of BOT [21], FSS has become increasingly required by patients with fertility requirements. For patients who were infertile after conservative surgery, ART is an option for improving pregnancy rate, although the impact of ART in such cases remains unclear.

It had been suggested that the use of ART can increase the risk of ovarian borderline tumors [19], and a previous study reported a potential link between the occurrence of ovarian tumors and infertility and ovulation induction drugs [22]. A cohort study of 3837 women by Rossing et al. found that the long-term use of clomiphene (> 12 months) may increase the risk of ovarian epithelial borderline or malignancy, especially in non-pregnant women [14]. In a case-controlled study, Ness et al. reported that the risk of developing ovarian malignancies was not related to the use of ART, but rather, the cause of infertility [23]. Other studies have suggested that the induction of ovulation can promote the occurrence of ovarian borderline epithelial tumors [10, 11, 13, 14, 23]. Although the pathological mechanisms involved remains unclear, some studies have suggested that these effects were more likely to be caused by changes in hormone levels than gene levels [24]. Leeuwen [17] performed a retrospective cohort study of 19,146 patients treated with IVF and 6006 patients without IVF treatment, but with poor fertility, and compared the two groups in regards to the incidence of ovarian malignancies (including BOT) during follow-up. During the 15-year study period, the incidence of borderline ovarian tumors in the IVF group increased compared with those without IVF treatment [normalized incidence (standardized incidence rate [SIR] = 1.76); 95% CI = 1.16–2.56)], and the incidence of ovarian cancer did not increase significantly. When the follow-up time exceeded 15 years, the incidence of ovarian cancer in the IVF group was 3.54, while the incidence of ovarian cancer in the non-IVF group did not change significantly. At same time, the incidence of borderline ovarian tumors in the two groups did not increase significantly. In our study, we also found that ART was an independent risk factor for the recurrence of BOT. Of the patients who received ART, the mean number of retrieved oocytes in the recurrence group (14.00 ± 9.744) was higher than the mean number of retrieved oocytes in the non-recurrence group (10.80 ± 9.041). We believe that the higher the number of IVF cycles, the greater the cumulative dose of ovulation-inducing drugs. There was a close relationship between ART and the recurrence of BOT in response to an increased rate of repeated repair and damage to ovarian epithelial cells. In addition, repeated needle aspiration for patients with ovarian borderline tumors might increase the possibility of tumor metastasis.

However, some previous authors considered that ART drugs have little to do with the occurrence of ovarian borderline tumors. For example, Bjørnholt et al. conducted a retrospective pathological cohort study of 96,545 women with fertility problems between 1963 and 2006. These authors found that BOT was associated with the use of progesterone and the cumulative amount of progesterone, independent of other ART drugs [25]. Such findings differed from those observed in the present study. One possible reason for this is that the subjects were different. Our study focused more on the relationship between recurrent BOT and ART drugs, not the primary BOT. In another paper, Park et al. [26] performed 10 ART cycles in five patients with BOT who had undergone fertility preservation surgery and found the clinical pregnancy rate, transplant rate and live birth rate were 50% (4/8), 31.6% (6/19) and 50% (4/8), respectively. Thus, IVF can be applied to BOT patients who are infertile after conservative surgery, and in particular, early BOT patients. There are also related case reports that report no recurrence of tumors in patients who underwent adjuvant reproductive therapy after fertility preservation surgery during a follow-up period of 2.5 to 5 years [27–30]. However, Beiner et al. [31] monitored seven patients with ovarian epithelial borderline tumors who underwent ART after fertility preservation surgery and found that the median follow-up time from the first postoperative ART cycle to the end of follow-up was 50 months. Four of the patients involved in this earlier study relapsed. Of those, two patients relapsed before ART, while the others relapsed after ART. All relapsed patients had the same pathological type as their initial tumor, but still chose conservative surgery. The results of this earlier study were similar to those of our present study in that the use of ART drugs might increase the risk of BOT recurrence.

Previous literature shows that the rate of recurrence in BOT patients after conservative treatment was higher for cystectomy than for unilateral adnexectomy. Most recurrences occurred in the remaining ovary. These earlier studies suggested that recurrence might be related to ruptures of the lesion or the resected margin of the cystectomy specimen [32, 33]. However, Yinon et al. [34] found that there was no significant difference in the rate of recurrence, when compared between UC and USO (22.7% vs. 27.5%, P = 0.8). For young women who want to retain fertility, it is recommended to perform fertility preservation surgery. The incidence of spontaneous pregnancy is about 50% [35]. Although the risk of recurrence was significantly associated with fertility preserving surgery, it had no effect on the survival rate of patients. Most recurrent diseases are non-invasive in nature and can be treated by conservative surgery. Therefore, the increase of recurrence rate after conservative surgery does not seem to affect the survival of patients [36]. In addition, pregnancy itself may not increase the risk of postoperative recurrence in BOT patients [37].

In our study, bilateral lesions significantly increased the risk of recurrence, as determined by our multivariate analysis. The recurrence rate of patients undergoing BC (16.67%) was higher than those with UA + CC (10.87%), although there was no statistical significance in the rate of recurrence between these two surgical types (P > 0.05). This showed that further research is now required to investigate the potential effect of surgery type.

The precise reason for infertility in BOT patients following conservative treatment remains unclear. The causative factors for infertility might involve a low rate of pregnancy before surgery or the harm caused by conservative surgical treatment. The patients selected in this study had no history of infertility prior to surgery. We found that conservative treatment, with comprehensive staged surgery, might be related to the postoperative natural pregnancy rate. Prat J [38] suggested that staged surgery was more prone to pelvic adhesions than the non-staged surgery. In other words, non-staged surgery better preserved fertility. In another study, Tao et al. [39] reported no statistical difference between surgical staging and postoperative pregnancy rate. Whether the extended range of comprehensive staging surgery would increase the infertility rate of patients requires further study. In our present study, the rate of IVF increased significantly in patients with surgical staging compared to patients with non-staging treatment (40% vs. 18.8%), although there was no significant difference between the two groups (P > 0.05). Due to the small sample size and short follow-up time of this study, it is now necessary to expand sample size and carry out assessments with long-term follow-up. On the other hand, we found that the rate of pregnancy before BOT was significantly higher in the non-ART treatment group than in the ART treatment group (0.64 ± 1.061 vs. 0.20 ± 0.414, P = 0.002). This means that the pregnancy rate before BOT was low, and that the chance of facing ART treatment would therefore increase in the future. This finding was consistent with clinical observations in that people with ART treatment usually have poor pregnancies and low pregnancy rates.

We also found that excessive intraoperative blood loss increased the chance of future ART treatment (128.00 ± 81.70 vs. 76.35 ± 80.90 mL, P = 0.03). However, we have not been able to draw a logical explanation from our clinical experience. We speculate that patients with greater intraoperative blood loss show an increased risk of postoperative adhesions, which therefore, leads to an increased risk of infertility and an increased chance of ART treatment after the surgery.

In conclusion, our data indicate that the postoperative use of ART in patients with BOT significantly increases the recurrence rate. For patients who must use ART to treat infertility, the risk of recurrence should be discussed carefully with the patients involved. However, we also observed that ART does not significantly affect the overall survival rate of patients, and often the recurrence pathology is the same as the original tumor. Therefore, ART in such patients should be individualized, and involve close follow-up.

## Data Availability

The data are available from the corresponding author on reasonable request.
